# Functional alleles of the flowering time regulator *FRIGIDA *in the *Brassica oleracea *genome

**DOI:** 10.1186/1471-2229-12-21

**Published:** 2012-02-14

**Authors:** Judith A Irwin, Clare Lister, Eleni Soumpourou, Yanwen Zhang, Elaine C Howell, Graham Teakle, Caroline Dean

**Affiliations:** 1 Department of Cell and Developmental Biology, John Innes Centre, Norwich Research Park, Norwich NR4 7UH, UK; 2 School of Biosciences, University of Birmingham, Birmingham B15 2TT, UK; 3 School of Life Sciences, University of Warwick, Wellesbourne CV35 9EF, UK

**Keywords:** FRIGIDA, Flowering time, vernalization, synteny, *Brassica oleracea*, *Arabidopsis thaliana*

## Abstract

**Background:**

Plants adopt different reproductive strategies as an adaptation to growth in a range of climates. In *Arabidopsis thaliana FRIGIDA *(*FRI*) confers a vernalization requirement and thus winter annual habit by increasing the expression of the MADS box transcriptional repressor *FLOWERING LOCUS C *(*FLC*). Variation at *FRI *plays a major role in *A. thaliana *life history strategy, as independent loss-of-function alleles that result in a rapid-cycling habit in different accessions, appear to have evolved many times. The aim of this study was to identify and characterize orthologues of *FRI *in *Brassica oleracea*.

**Results:**

We describe the characterization of *FRI *from *Brassica oleracea *and identify the two *B. oleracea FRI *orthologues (*BolC.FRI.a *and *BolC.FRI.b*). These show extensive amino acid conservation in the central and C-terminal regions to FRI from other Brassicaceae, including *A. thaliana*, but have a diverged N-terminus. The genes map to two of the three regions of *B. oleracea *chromosomes syntenic to part of *A. thaliana *chromosome 5 suggesting that one of the *FRI *copies has been lost since the ancient triplication event that formed the *B. oleracea *genome. This genomic position is not syntenic with *FRI *in *A. thaliana *and comparative analysis revealed a recombination event within the *A. thaliana FRI *promoter. This relocated *A. thaliana FRI *to chromosome 4, very close to the nucleolar organizer region, leaving a fragment of *FRI *in the syntenic location on *A. thaliana *chromosome 5. Our data show this rearrangement occurred after the divergence from *A. lyrata*. We explored the allelic variation at *BolC.FRI.a *within cultivated *B. oleracea *germplasm and identified two major alleles, which appear equally functional both to each other and *A. thaliana FRI*, when expressed as fusions in *A. thaliana*.

**Conclusions:**

We identify the two *Brassica oleracea FRI *genes, one of which we show through *A. thaliana *complementation experiments is functional, and show their genomic location is not syntenic with *A. thaliana FRI *due to an ancient recombination event. This has complicated previous association analyses of *FRI *with variation in life history strategy in the *Brassica *genus.

## Background

The switch to reproductive development is a fundamental process in the plant life cycle. The molecular mechanisms underlying this developmental transition have been extensively studied in *Arabidopsis thaliana*. An integrated network of environmentally responsive genetic pathways converge on a common set of targets to quantitatively regulate the genes required to switch the apical meristem from a vegetative to a floral state [1-3]. One important environmental cue is prolonged cold, which accelerates flowering in a process termed vernalization and aligns pollination and seed set with the favourable conditions of spring. Variation in requirement for vernalization exists in many plant species and this influences life history strategy with plants requiring vernalization adopting a perennial, biennial or winter annual habit in contrast to summer annuals, which flower in the first growing season. This is in contrast to other species that are more reliant on photoperiodic signals or endogenous cues e.g. rice [4]. The significant fitness consequences of flowering time variation, demonstrated in annual [5,6] and perennial plants [7], have most likely contributed to the evolution of the extensive variability in flowering time control. Flowering also influences the pattern of growth throughout the seasons and affects many agronomic characters including the quantity and quality of crop production. This is particularly apparent in cultivated brassicas, where variation in the flowering process has been selected to produce a diverse array of economically important morphological forms.

A major determinant in the variation of vernalization requirement in *A. thaliana *is allelic variation at *FRIGIDA *(*FRI*) [8-11]. FRI represses flowering by promoting the expression of the floral repressor *FLOWERING LOCUS C *(*FLC*) [12,13]. Vernalization acts antagonistically to FRI and accelerates flowering by down-regulating *FLC*. A number of rapid-cycling variants of *A. thaliana *that do not need vernalization were found to have arisen through loss of function of *FRI*, an evolutionary step that has occurred multiple times [8,9,11,14]. Parallel evolution through allelic variation at a common target has been found in other organisms [15]. It was therefore interesting to ask whether a similar evolutionary step has occurred in other plant species. Many other species do show variation in vernalization requirement and it is an important agronomic trait in many major crops. For example, in *B. oleracea *(horticultural brassicas) vernalization-requiring biennials are represented by cabbage and Brussels sprouts, with summer annual crops including some calabrese and cauliflower cultivars. Orthologues of *FRI *have been identified in *A. lyrata *[16], *Capsella *species [17] and the halophyte *Thellungiella halophila *[18] within the Brassicaceae, and more broadly in *Medicago truncatula, Lotus japonicus, Vitis vinifera *[19], *Populus balsamifera *[20] and *Oryza sativa *[21]. To date natural variation in vernalization requirement has been associated with *FRI *polymorphism in *A. lyrata *[8] and allelic variation in one orthologue in *Brassica napus *(*BnaA.FRI.a*) has been associated with flowering time variation [22].

We are interested in understanding the molecular basis of variation in flowering time and vernalization requirement in horticultural brassicas. Genetic information from *A. thaliana *can generally be applied to Brassica species because of their evolutionary relatedness. The *Arabidopsis *and *Brassica *genera are in the same family (Brassicaceae) with *B. oleracea *thought to have arisen from a triplication of an ancestral genome similar to that of *Arabidopsis *[23-26]. Genetic information on the control of flowering in *Arabidopsis *can be applied to Brassica species because of the colinearity of the *Arabidopsis *and *Brassica *genomes [23,27,28]. This has been used to infer candidate genes that might account for QTL underlying flowering time and other variation [22,29,30]; however, in some instances it can be misleading [31]. Here, we identify the two *FRI *genes in the *B. oleracea *genome and map their genomic locations. We also explored allelic variation at one of the *FRI *loci in cultivated *B. oleracea *germplasm. These new data will provide the necessary information to elucidate how general a role *FRI *plays in life history variation in the Brassicaceae.

## Results and discussion

### Two *FRI *genes are present in the *Brassica oleracea *genome

**Figure 1:**
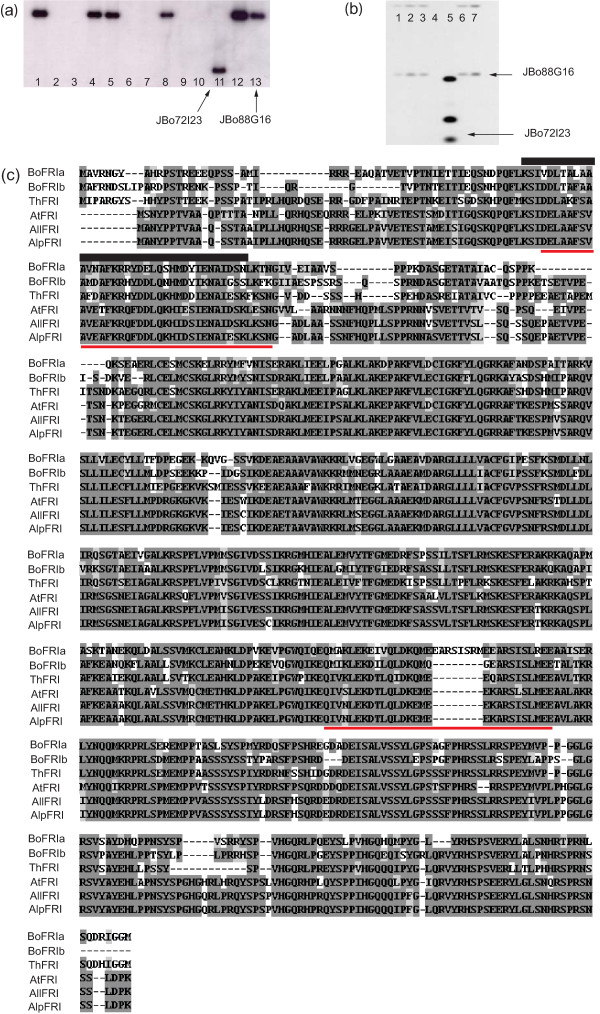
**Cloning *BoFRIa *and *BoFRIb***. (**a**) Southern analysis of A12 BACs identified by colony hybridisation and probed with *AtFRI*. Lanes 1, 4, 5, 8, 11, 12 and 13 contain clones that show homology to *FRIGIDA*. BACs in lanes 2, 3, 6, 7, and 10 do not cross hybridise (**b**) HindIII digest of six BACs probed with conserved region from exons 2 and 3 of *BoFRIa*. Lane 5 contains JBo72I23 from which *BoFRIa *was sequenced. Lane 7 contains JBo88G16. Lanes 1, 2, 3 and 6 contain four further BACs showing the same hybridization pattern as JBo88G16. Note the intensity of the hybrization is indicative of the sequence divergence between BoFRIa and BoFRIb (See 1c) (**c**) Comparison of the protein sequences of BoFRIa and BoFRIb with other members of the FRI sub-family. From top to bottom they are *Brassica oleracea *BoFRIa, *Brassica oleracea *BoFRIb, *Thelliungiella halophila *ThFRI, Arabidopsis *thaliana *AtFRI *Arabidopsis lyrata *ssp *lyrata *AllFRI and *Arabidopsis lyrata *ssp *petraea *AlpFRI. The N-terminal domain containing the conserved region of 37 amino acids (indicated by solid bar) that defines copies of FRIGIDA from other members of the FRI superfamily [17]. The coiled-coil domains are indicated by the red lines.

The *BoFRI *genes were isolated from the JBo BAC library of the *B. oleracea *Chinese kale genotype A12DHd [32] through hybridization with an *A. thaliana FRI *genomic clone. From seven positive BAC clones two that showed distinct *FRI *hybridization patterns (JBo72I23 and JBo88G16, Figure 1a, b) were selected for sub-cloning. Analysis of these confirmed they carried different *Brassica *paralogues designated *BolC.FRI.a *and *BolC.FRI.b *[33] and referred to hereafter as *BoFRIa *and *BoFRIb *[GenBank JN191450 and JN191449]. As in other species, *BoFRIa *and *BoFRIb *contain three exons encoding predicted open reading frames (ORFs) of 594 and 585 residues respectively (Figure 1c). BoFRIa contains two coiled-coil domains, typically involved in protein oligomerisation (as predicted by COILS http://www.ch.embnet.org/software/COILS_form.html[34]), very similar to the predicted structure of the *A. thaliana *FRI (AtFRI) [8,35]. In contrast, BoFRIb is predicted to contain only one coiled-coil domain in the C-terminal region as was found to be the case for two of the four FRI identified in *B. napus *[22].

AtFRI is the original member of a family of seven proteins in *A. thaliana *which, apart from the two predicted coiled-coil domains, show no homology with any other proteins and whose function has yet to be determined. Recent analysis of the FRI protein family [19] identified a conserved core central domain. Outside of this domain significant variation is observed that allows the FRI family to be subdivided into five distinct groups. AtFRI and its orthologues in other species are defined by a conserved region of 37 amino acids in the N-terminal region of the protein. The BoFRI proteins we describe here contain this conserved 37 amino acid region reinforcing the view they are FRI orthologues; however, the amino acids either side of this region show lower homology to AtFRI (Figure 1c). This region includes much of the first predicted coiled-coil in BoFRIa. Variations in this domain in BoFRIb result in the loss of a predicted coiled-coil, emphasising a possible functional significance for the amino acid polymorphisms in this region. A similar degree of divergence from AtFRI is found in the N-terminal region of an orthologue of FRI isolated from the halophyte *T. halophila *and in four orthologues of FRI identified in *B. napus *[18,22]. By contrast, there is extensive amino acid conservation between BoFRIa, BoFRIb and AtFRI in the central and C-terminal regions (Figure 1c). Transgenic analysis of the functional domains of AtFRI in *A. thaliana *where the N or C terminus was deleted revealed that the N-terminal region was less important for function [19], perhaps explaining the high degree of divergence observed.

### The *BoFRI *genes map to regions that are non-syntenic with *A. thaliana FRI*

**Figure 2:**
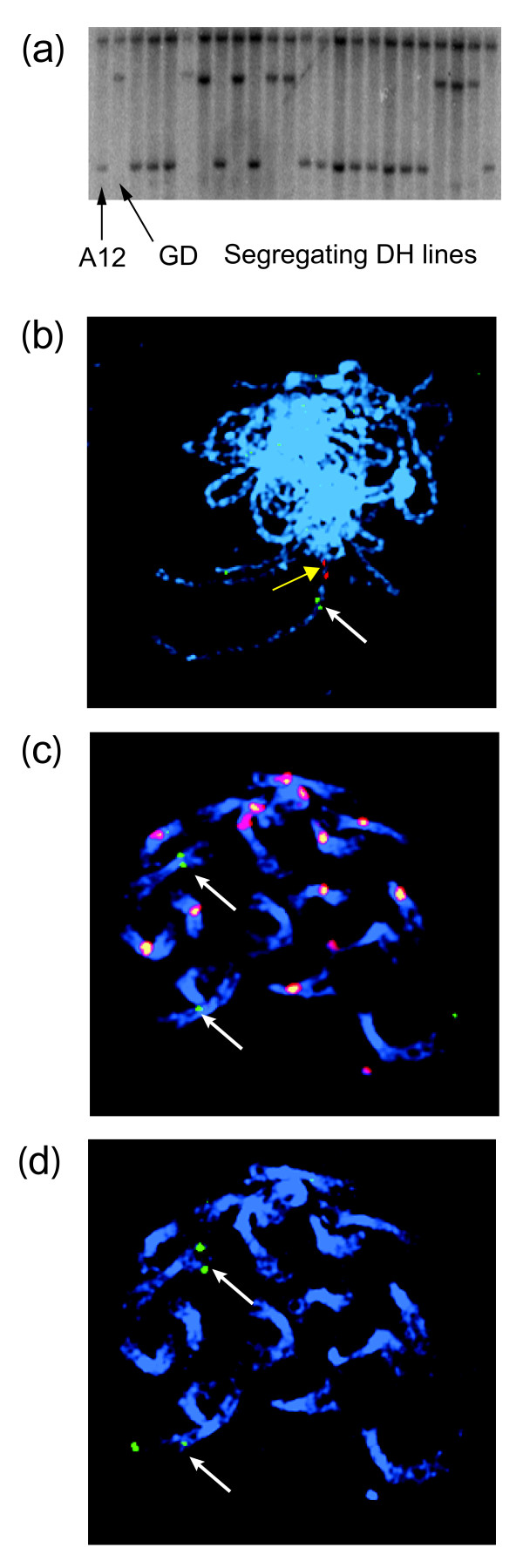
**Mapping *BoFRIa *and *BoFRIb***. (**a**) A12DHdxGDDH33 mapping population probed with conserved region from exons 2 and 3 of *BoFRIa*. Two loci are identified; one monomorphic (upper band) and a second segregating with the two parental alleles (lower two bands). (**b**) Meiotic pachytene spread with JBo72I23 (*BoFRIa*, green, white arrow) hybridizing to C3 between the telomere and JBo62M08 (red, yellow arrow). (**c**) and (**d**) JBo88G16 (*BoFRIb*) hybridizes to the short arm of C9: (**c**) mitotic metaphase with JBo88G16, (green, arrows), BoB061G14 and 45S rDNA (red), (**d**) reprobe of (**c**) with JBo32J18 (green, arrows), a marker for C9.

A genomic fragment including exon 2, intron 2 and exon 3 of *BoFRIa *(and showing a high level of conservation in *BoFRIb *and *AtFRI*) was hybridized to mapping filters from two *B. oleracea *mapping populations: Chinese kale × calabrese (var. *alboglabra *x var. *italica; *A12DHdxGDDH33, [36]; Figure 2a) and cauliflower × Brussels sprout (var. *botrytis *x var. *gemmifera*; N × G [37]). RFLPs for one of the two *BoFRI *loci segregated in the A12DHdxGDDH33 mapping population that allowed this locus to be mapped to 39.5 cM on linkage group C3 of the *B. oleracea *genome. The locus mapping to C3 was identified as *BoFRIa *by fluorescence in situ hybridization (FISH) with BAC JBo72I23, from which *BoFRIa *was originally sequenced (Figure 2b). JBo88G16 was located on the short arm of chromosome C9 by FISH (Figure 2c, d). Therefore, the second locus, *BoFRIb*, was on linkage group C9. Two further BACs showing the same restriction pattern as JBo88G16 (Figure 1b) hybridized to the same location on C9 (data not shown). These results confirm that the *B. oleracea *genome contains two orthologues.

Comparative analysis of the *Brassica *and *A. thaliana *genomes has shown that the chromosomal regions of C3 and C9 to which the two *BoFRI *loci have been mapped are syntenic to a region of *A. thaliana *chromosome 5 and not to the top of *A. thaliana *chromosome 4, where *AtFRI *(At4g00650) is located [23]. This region of chromosome 5 includes a number of genes known to be involved in the control of flowering including *FLC, FY *and *CONSTANS *(*CO*). Several QTL studies have found loci for flowering time variation mapping to this genomic region in a number of *Brassica *populations including *B. oleracea *[38-41], *B. rapa *[42-46]; *B. nigra *[47,48] and *B. napus *[29,49]. The mapping we have undertaken reveals the proximity of *BoFRI *not only to *BoFLC*, but also *BoFY *and *BoCO; *other flowering time genes that have been mapped previously. The sequences of *BoFRIa *and *BoFRIb *further allow us to identify which of the four orthologues of *AtFRI *recently identified in *B. napus *[22] are the two C genome copies. The four copies of *FRI *were designated *BnaA.FRI.a*, and *BnaX.FRIb-d*. Comparison of the amino acid sequences of these proteins with BoFRIa and BoFRIb suggest that BnaX.FRI.d is the orthologue of BoFRIa and the C genome homoeologue of BnaA.FRI.a. This conclusion is further supported by the fact that *BnaA.FRI.a *was mapped to a region of A3 homoeologous to the region of C3 where we have mapped *BoFRIa*. Comparison of the amino acid sequence of BnaX.FRI.c shows it to be identical to that of BoFRIb. BnaX.FRI.c appears most similar to BnaX.FRI.b and is therefore likely to be the A genome homoeologue of BoFRIb in *B. napus*.

### A recombination event specific to the *A. thaliana *lineage has relocated the *FRIGIDA *gene to the top of chromosome 4

**Figure 3:**
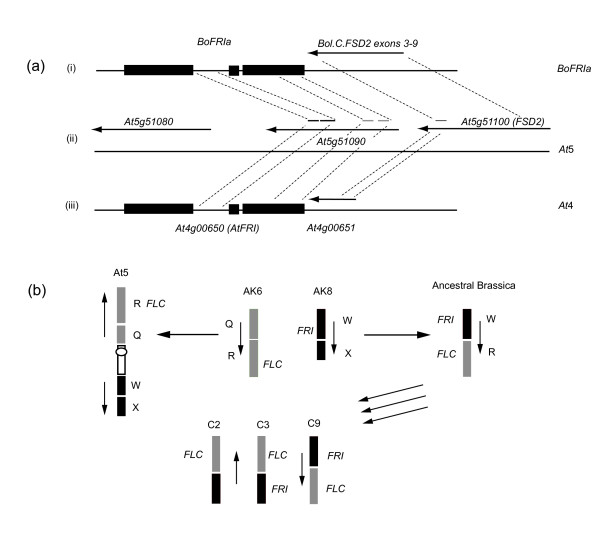
***BoFRIa *and *BoFRIb *map to regions of the *B. oleracea *genome that are non-syntenic with A. thaliana**. (**a**) Comparison of *BoFRIa *genomic clone with annotated regions of *A. thaliana *chromosomes 4 and 5 represented in the 5' to 3' orientation. Solid black rectangles correspond to the three exons that make up *FRI*. Arrows indicate the orientation of other genes. (i) *BoFRIa*, genomic clone. (ii) Region of *A. thaliana *chromosome 5 containing an iron superoxide dismutase (*FSD2, At5g51100*) and *At51090 *showing homology to *FRI *intron 1 and exon 3. (iii) Region of *A. thaliana *chromosome 4 containing *AtFRI* and *At4g400651 *showing homology to exon 9 of *FSD2. (**b**) *Proposed derivation of *A. thaliana *and *B. oleracea *linkage groups from ancestral karyotype (AK6 and AK8) as suggested in [53] showing the position of *FRI *and *FLC*. The rearrangement of ancestral blocks W and R results in orthologues of *FRI *and *FLC *being brought into close proximity in the ancestral Brassica genome. This region is present on chromosomes C2, C3 and C9 of *B. oleracea *and is located in regions of the *B. oleracea *genome showing synteny to *A. thaliana *chromosome 5. *BoFRIa *is located on C3 and *BoFRIb *on C9. The copy of *FRI* on C2 has been lost.

In *A. thaliana*, the *AtFRI *locus is located at the top of chromosome 4. However, it has previously been reported that the orthologue of *FRI *in *A. lyrata *maps to linkage group 8 [50,51]. This linkage group is orthologous to the lower arm of *A. thaliana *chromosome 5 [50,52,53]. Interestingly, an annotated gene model in this region of *A. thaliana *chromosome 5 (*At5g51090*) shows a high degree of homology to *AtFRI*, containing parts of intron 1 and exon 3 but lacking other parts of the coding region, thus it may be a pseudogene [50]. Genevestigator data suggest *At5g51090 *is expressed at very low levels, supporting this hypothesis [54]. Downstream, in the opposite orientation, is *At5g51100*, encoding an iron superoxide dismutase and the *BoFRIa *BAC clone contains 3' sequence showing homology to exons 3-9 of this *A. thaliana *chromosome 5 gene (Figure 3a).

Synteny has been studied extensively in the Brassicaceae genomes due to its potential for gene identification and marker development. *Arabidopsis *and *Brassica *are thought to have diverged about 43 Mya with a triplication of an ancestral genome (similar to that of *Arabidopsis*) occurring approximately 23 Mya and giving rise to modern day diploid Brassica genomes [55]. *A. thaliana *and *A. lyrata *are thought to have diverged around 13 Mya, with a reduction in chromosome number, from the ancestral karyotype of n = 8 (as represented in *A. lyrata*) to the derived state in *A. thaliana *of n = 5 [24,55,56]. The ancestral karyotype of the Brassicaceae is proposed to be eight chromosomes composed of 24 conserved chromosomal blocks [57]. These blocks can be rearranged to model the genome structure of *A. thaliana, A. lyrata *and the modern day diploid *Brassicas *[24]. Thus the genomic composition of the nine chromosomes (C1-C9) of *B. oleracea *and ten chromosomes (A1-A10) of *B. rapa *can be related to both the ancestral karyotype and the *A. thaliana *genome.

The ancestral genomic blocks QR and WX from chromosomes 6 and 8 respectively of the ancestral karyotype, and today represented by *A. lyrata *[24], have been recombined in the ancestral *Brassica *genome prior to triplication, leading to the block WR being represented three times in the *B. rapa *genome on A2, A3 and A10 [25,26]. The paralogous regions of *B. oleracea *are on C2, C3 and C9 (Figure 3b). This rearrangement brings orthologues of *FLC *(block R) and *FRI *(block W) together on these chromosomes. Thus in *A. lyrata *and *B. oleracea, FRI *maps quite closely to *VIN3 *(also in block W and required for vernalization [58], as well as its major target *FLC *(block R; Figure 3b). *BoFRIa *mapping to C3 and *BoFRIb *to C9 thus represent two of the three syntenic regions. The third paralogue of *FRI *appears to have been lost from C2 during *B. oleracea *evolution; such gene loss is not uncommon [59]. This is in contrast to the current location of *FRI *at top of chromosome 4 in *A. thaliana *that shows homology to block O from chromosome 6 of the ancestral karyotype.

The data we present here suggest that the chromosomal rearrangements that occurred during the evolution of the ancestral Brassicaceae genome into *A. thaliana *included a recombination/rearrangement event that relocated a genomic region containing *AtFRI *to a position near the distal end of the short arm of chromosome 4, close to the nucleolar organiser region, leaving a non-functional remnant in the genomic position on chromosome 5 that is syntenic with *FRI *in the other Brassicaceae (*A. lyrata, B. oleracea; *[25,52]).

### Two common alleles exist for *BoFRIa *in diverse genotypes of *B. oleracea*

The original sequences of *BoFRI*a and *BoFRIb *were obtained from A12DHd, one of the parents of the mapping population used in [38,39]. These studies mapped QTL for flowering time on C3 in the region where we have mapped *BoFRIa*. We therefore sequenced 650 bp from exon 1 of *BoFRIa *from the other parent of this population, GDDH33 (data not shown). The GDDH33 sequence showed two amino acid substitutions (A118V and Q125E) compared to A12DHd. Thus the parents of this population are carrying different alleles of *BoFRIa *and it is possible that variation at *BoFRIa *is contributing to variation in flowering time in this population. Single amino acid substitutions have also been identified in alleles of *BnaA.FRI.a *sequenced from the parents of the Tapidor × Ningyou7 (TN) mapping population from *B. napus *and mapped to a region underlying a QTL for flowering time variation [22].

We sequenced *BoFRIa *and *BoFRIb *for two additional genotypes of *B. oleracea italica*. The A12DHd reference sequence is derived from a BAC clone of a Chinese kale, *B. oleracea alboglabra*, which flowers after 8 weeks [38] and can be considered a rapid-cycling type, not requiring vernalization. We therefore selected two additional genotypes of broccoli (*Brassica oleracea italica*); E1, which has a facultative vernalization response, flowering earlier following a period of cold, but which matures in October/November (Autumn) of the year of planting and E8 which has an obligate vernalization requirement and matures in April/May (Spring) of the following year. BoFRIb is highly conserved between the three genotypes with only 5 amino acid substitutions (D6G, K20Q, Q372K and R532W in E1 and N105T in E8). The sequencing of *BoFRIa *in these genotypes identified a polymorphic region in exon 1 that included two deletions of seven and three amino acids in E8 relative to E1, either side of the conserved block of 37 amino acids that defines the FRI proteins, (Figure 1c). Thirteen non-synonymous and 12 synonymous substitutions differentiate the A12DHd and E1 BoFRIa alleles from the E8 allele. We therefore designated the E1 and E8 *BoFRIa *alleles as *BoFRIa-1 *and *BoFRIa-4 *respectively [GenBank JN191393, JN191392].

**Figure 4:**
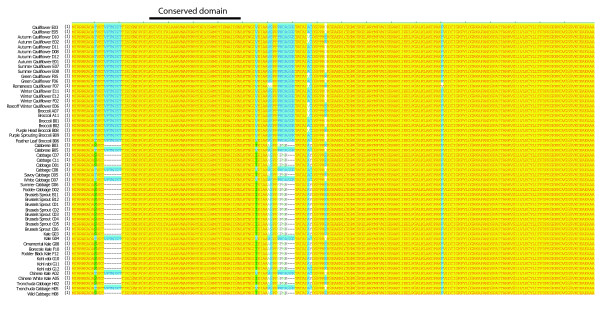
**Comparison of first 160 amino acids of BoFRIa from 55 BolDFS genotypes**. Protein comparison of BoFRIa from cultivated genotypes of *B. oleracea *listed by crop type. The plate co-ordinates refer to those listed in Table 1. The conserved region of 37 amino acids that defines FRI from other members of the FRI superfamily is delineated by the horizontal black bar.

We focused our subsequent analysis on *BoFRIa *as this showed most polymorphism and extended our analysis to include 55 genotypes from the cultivated *B. oleracea *Diversity Foundation Set developed at the University of Warwick (BolDFS, King et al. http://www.brassica.info/resource/plants/diversity_sets.php; [60] Table 1). A 650 bp region of *BoFRIa *covering the exon 1 polymorphic region containing the two deletions was sequenced (Table 1, GenBank JN191394-191448). We identified six *BoFRIa *alleles within this subset of 55 genotypes from the BolDFS. These can be divided into two groups; *BoFRIa 1-3 *and *BoFRIa 4-6 *where alleles in the second group include the seven amino acid and three amino acid deletions. The *BoFRIa-1 *and *BoFRIa-4 *alleles were the most common within the 55 genotypes studied. In addition the two deletions identified in *BoFRIa-4 *always co-occurred and were found at high frequency together with a small number of non-synonymous nucleotide polymorphisms. The two deletions, which had been found in the late-flowering broccoli, were over-represented in *B. oleracea *vegetable types such as Brussels sprouts and kohl rabi with a winter annual or biennial habit, usually grown for consumption of their vegetative rather than floral forms (Table 1, Figure 4). Interestingly *BnaX.FRI.d *from the *B. napus *winter variety Express [22], which we have identified here as the C genome homologue of *BoFRIa *in *B. napus *has both of the deletions identified in the *BoFRI 4-6 *class of alleles that are overrepresented in brassica vegetable types with a winter annual or biennial habit. On closer examination BnaX.FRI.d was found to have the same amino acid sequence as BoFRIa-5 and is also present in the European winter type and Chinese semi-winter type parental lines of the TN mapping population [22].

**Table 1:** Amino acid polymorphisms in BoFRIa from cultivated genotypes of *Brassica oleracea*

Line	Accession name	Crop type	Crop group	Ref	origin	cult/landrace/DH	32	36-42	97	102	103	106	107	108	109	110-112	118	119	125	172	*BoFRIa *Allele
SIR5a	Siria DH line 5a	Cauliflower	Cauliflower	E03	**N/A**	**DH**	**T**		**V**	**V**	**S**	**P**	**K**	**D**	**A**		**A**	**C**	**Q**	**F**	**1**

HRIGRU002838	ALGROMAJO NO 2	Cauliflower	Cauliflower	E05	**NL**	**cultivar**	**T**		**V**	**V**	**S**	**P**	**K**	**D**	**A**		**A**	**C**	**Q**	**F**	**1**

HRIGRU004832	TOSCANO	Autumn cauliflower	Cauliflower	D10	**ITA**	**cultivar**	**T**		**V**	**V**	**S**	**P**	**K**	**D**	**A**		**V**	**C**	**E**	**F**	**2**

HRIGRU005458	DI JESI	Autumn cauliflower	Cauliflower	E02	**ITA**	**cultivar**	**T**		**V**	**V**	**S**	**P**	**K**	**D**	**A**		**V**	**C**	**E**	**F**	**2**

HRIGRU006254	TASMAN	Autumn cauliflower	Cauliflower	D11	**AUS**	**cultivar**	**T**		**V**	**V**	**S**	**P**	**K**	**D**	**A**		**A**	**C**	**Q**	**F**	**1**

HRIGRU004239	CANBERRA	Autumn cauliflower	Cauliflower	D08	**AUS**	**cultivar**	**T**		**V**	**V**	**S**	**P**	**K**	**D**	**A**		**A**	**C**	**Q**	**F**	**1**

HRIGRU008558	GIGANTE DI NAPOLI NATALINO *	Autumn cauliflower	Cauliflower	D12	**ITA**	**N/A**	**T**		**V**	**V**	**S**	**P**	**K**	**D**	**A**		**A**	**C**	**Q**	**F**	**1**

HRIGRU006797	SOFIA	Autumn cauliflower	Cauliflower	E01	**SP**	**cultivar**	**T**		**V**	**V**	**S**	**P**	**K**	**D**	**A**		**A**	**C**	**Q**	**F**	**1**

HRIGRU004814	BIANCO NAPOLETANE NATALINO	Summer cauliflower	Cauliflower	E07	**ITA**	**cultivar**	**T**		**V**	**V**	**S**	**P**	**K**	**D**	**A**		**A**	**C**	**Q**	**F**	**1**

HRIGRU004991	ALL THE YEAR ROUND	Summer cauliflower	Cauliflower	E08	**UK**	**N/A**	**T**		**V**	**V**	**S**	**P**	**K**	**D**	**A**		**A**	**C**	**Q**	**F**	**1**

HRIGRU004847	VERDE DI MACERATA	Green caulilfower	Cauliflower	F05	**ITA**	**N/A**	**T**		**V**	**V**	**S**	**P**	**K**	**D**	**A**		**V**	**C**	**E**	**F**	**2**

HRIGRU004850	DI ALBENGA	Green caulilfower	Cauliflower	F06	**ITA**	**cultivar**	**T**		**V**	**V**	**S**	**P**	**K**	**D**	**A**		**A**	**C**	**Q**	**F**	**1**

HRIGRU004861	ROMANESCO NATALINO	Romanesco cauliflower	Cauliflower	F07	**ITA**	**cultivar**	**T**		**V**	**A**	**P**	**P**	**K**	**D**	**A**		**V**	**C**	**E**	**V**	**3**

HRIGRU002891	ST MALO HALF HATIF	Winter cauliflower	Cauliflower	E11	**FRA**	**cultivar**	**T**		**V**	**V**	**S**	**P**	**K**	**D**	**A**		**A**	**C**	**Q**	**F**	**1**

HRIGRU004492	WINTER ROSCOFF	Winter cauliflower	Cauliflower	E12	**IRE**	**landrace**	**T**		**V**	**V**	**S**	**P**	**K**	**D**	**A**		**A**	**C**	**Q**	**F**	**1**

HRIGRU006230	LATE QUEEN	Winter cauliflower	Cauliflower	F02	**IND**	**cultivar**	**T**		**V**	**V**	**S**	**P**	**K**	**D**	**A**		**A**	**C**	**Q**	**F**	**1**

ROS152b	Roscoff type F1 DJ1356 DH line 152b	Roscoff winter cauliflower	Cauliflower	E06	**N/A**	**DH**	**T**		**V**	**V**	**S**	**P**	**K**	**D**	**A**		**V**	**C**	**E**	**F**	**2**

Cor12b	Corvette DH line 12b	broccoli	Broccoli	A07	**N/A**	**DH**	**T**		**V**	**V**	**S**	**P**	**K**	**D**	**A**		**V**	**C**	**E**	**F**	**2**

CAL 18b	DH line Royal Sluis F1 RS71343 (DJ6546)	broccoli	Broccoli	A11	**N/A**	**DH**	**T**		**V**	**V**	**S**	**P**	**K**	**D**	**A**		**V**	**C**	**E**	**F**	**2**

HRIGRU002398	PICOLINI DI PALERMO	broccoli	Broccoli	B01	**ITA**	**cultivar**	**T**		**V**	**V**	**S**	**P**	**K**	**D**	**A**		**A**	**C**	**Q**	**F**	**1**

HRIGRU011802	MUGNULI	broccoli?	Broccoli	B02	**ITA**	**landrace**	**T**		**V**	**V**	**S**	**P**	**K**	**D**	**A**		**A**	**C**	**Q**	**F**	**1**

HRIGRU005276	CIMA VIOLETTA NATALINO	Purple head broccoli	Broccoli	B08	**ITA**	**cultivar**	**T**		**V**	**V**	**S**	**P**	**K**	**D**	**A**		**V**	**C**	**E**	**F**	**2**

HRIGRU003543	PURPLE SPROUTING LATE IMPROVED	Sprouting broccoli	Broccoli	B09	**UK**	**cultivar**	**T**		**V**	**V**	**S**	**P**	**K**	**D**	**A**		**A**	**C**	**Q**	**F**	**1**

HRIGRU005416	CAVOLO CAVOLINA RIZZA	Feather leaf broccoli	Broccoli	B06	**ITA**	**cultivar**	**T**		**V**	**V**	**S**	**P**	**K**	**D**	**A**		**A**	**C**	**Q**	**F**	**1**

HRIGRU004705	RAMOSO CALABRESE PRECOCE	Calabrese	Broccoli	B03	**ITA**	**cultivar**	**S**	**DEL**	**I**	**A**	**S**	**S**	**P**	**N**	**K**	**DEL**	**A**	**Y**	**E**	**F**	**4**

HRIGRU005425	CAVOLO BROCCOLO NATALINO	Calabrese	Broccoli	B05	**ITA**	**cultivar**	**T**		**V**	**V**	**S**	**P**	**K**	**D**	**A**		**A**	**C**	**Q**	**F**	**1**

BOH 85c	Bohmerwaldkohl DH line 85c	Cabbage	Cabbage	C07	**N/A**	**DH**	**S**	**DEL**	**I**	**A**	**S**	**S**	**P**	**N**	**K**	**DEL**	**A**	**Y**	**E**	**F**	**4**

HRIGRU005652	SHETLAND CABBAGE	Cabbage	Cabbage	C11	**UK**	**N/A**	**S**	**DEL**	**I**	**A**	**S**	**S**	**P**	**N**	**K**	**DEL**	**A**	**Y**	**E**	**F**	**4**

HRIGRU007833	LARGE BLOOD RED	Cabbage	Cabbage	D01	**IND**	**cultivar**	**S**	**DEL**	**I**	**A**	**S**	**S**	**P**	**N**	**K**	**DEL**	**A**	**Y**	**E**	**F**	**4**

HA 84a	Hawke DH line Ha84a	Cabbage	Cabbage	C08	**N/A**	**DH**	**T**		**V**	**V**	**S**	**P**	**K**	**D**	**A**		**A**	**C**	**Q**	**F**	**1**

HRIGRU004773	CAVOLO VERZA SAN GIOVANNI	Savoy cabbage	Cabbage	D05	**ITA**	**cultivar**	**T**	**DEL**	**I**	**A**	**S**	**S**	**P**	**N**	**K**	**DEL**	**A**	**Y**	**E**	**F**	**5**

HRIGRU011490	COUVE REPOLHO BACALAN	White cabbage	Cabbage	D07	**PORT**	**landrace**	**T**		**V**	**V**	**S**	**P**	**K**	**D**	**A**		**A**	**C**	**Q**	**F**	**1**

HRIGRU004771	CAVOLO CAPPUCCIO MEDIO NAPOLETANE	Summer cababge	Cabbage	D06	**ITA**	**cultivar**	**S**	**DEL**	**I**	**A**	**S**	**S**	**P**	**N**	**K**	**DEL**	**A**	**Y**	**E**	**F**	**4**

HRIGRU002574	CATTLE (EARLY DRUMHEAD)	Fodder cabbage	Cabbage	D02	**UK**	**cultivar**	**S**	**DEL**	**I**	**A**	**S**	**S**	**P**	**N**	**K**	**DEL**	**A**	**Y**	**E**	**F**	**4**

AC582	DH ex. Nym	Brussels sprout	Brussels sprout	B11	**N/A**	**DH**	**S**	**DEL**	**I**	**A**	**S**	**S**	**P**	**N**	**K**	**DEL**	**A**	**Y**	**E**	**F**	**4**

HRIGRU000342	EVESHAM GIANT	Brussels sprout	Brussels sprout	B12	**UK**	**cultivar**	**S**	**DEL**	**I**	**A**	**S**	**S**	**P**	**N**	**K**	**DEL**	**A**	**Y**	**E**	**F**	**4**

HRIGRU000605	WILHELMSBURGER	Brussels sprout	Brussels sprout	C01	**DEN**	**cultivar**	**S**	**DEL**	**I**	**A**	**S**	**S**	**P**	**N**	**K**	**DEL**	**A**	**Y**	**E**	**F**	**4**

HRIGRU002227	SANDA ROEM VAN CASTRICUM	Brussels sprout	Brussels sprout	C02	**UK**	**cultivar**	**S**	**DEL**	**I**	**A**	**S**	**S**	**P**	**N**	**K**	**DEL**	**A**	**Y**	**E**	**F**	**4**

HRIGRU002787	GROENENBOOM LATE SELECTION	Brussels sprout	Brussels sprout	C03	**NL**	**cultivar**	**S**	**DEL**	**I**	**A**	**S**	**S**	**P**	**N**	**K**	**DEL**	**A**	**Y**	**E**	**F**	**4**

HRIGRU005086	OLD BEDFORDSHIRE STOCK	Brussels sprout	Brussels sprout	C04	**UK**	**cultivar**	**S**	**DEL**	**I**	**A**	**S**	**S**	**P**	**N**	**K**	**DEL**	**A**	**Y**	**E**	**F**	**4**

HRIGRU006212	CAVOLO DI BRUXELLES MEZZO NANO	Brussels sprout	Brussels sprout	C05	**ITA**	**cultivar**	**S**	**DEL**	**I**	**A**	**S**	**S**	**P**	**N**	**K**	**DEL**	**A**	**Y**	**E**	**F**	**4**

HRIGRU008226	LOCAL SELECTION	Brussels sprout	Brussels sprout	C06	**BHUTAN**	**landrace**	**S**	**DEL**	**I**	**A**	**S**	**S**	**P**	**N**	**K**	**DEL**	**A**	**Y**	**E**	**F**	**4**

CGN14111	Butzo	Kale	Kale	G03	**N/A**	**N/A**	**S**	**DEL**	**I**	**A**	**S**	**S**	**P**	**N**	**K**	**DEL**	**A**	**Y**	**E**	**F**	**4**

HRIGRU006226	GIANT JERSEY KALE	Kale	Kale	G04	**UK**	**cultivar**	**T**		**V**	**V**	**S**	**P**	**K**	**D**	**A**		**A**	**C**	**Q**	**F**	**1**

HRIGRU009846	RED ON GREEN	Ornamental kale	Kale	G08	**JPN**	**cultivar**	**S**	**DEL**	**I**	**A**	**S**	**S**	**P**	**N**	**K**	**DEL**	**A**	**Y**	**E**	**F**	**4**

HRIGRU003598	WESTLAND WINTER VERDURA	Borecole kale	Kale	F10	**UK**	**cultivar**	**S**	**DEL**	**I**	**A**	**S**	**S**	**P**	**N**	**K**	**DEL**	**A**	**Y**	**E**	**F**	**4**

HRIGRU006210	CAVOLO NERO DI TOSCANA O *	Fodder black kale	Kale	F12	**ITA**	**cultivar**	**S**	**DEL**	**I**	**A**	**S**	**S**	**P**	**N**	**K**	**DEL**	**A**	**Y**	**E**	**F**	**4**

HRIGRU011183	PURPLE VIENNA	Kohl rabi	Kohl rabi	G10	**USA**	**cultivar**	**S**	**DEL**	**I**	**A**	**S**	**S**	**P**	**N**	**K**	**DEL**	**A**	**Y**	**E**	**F**	**4**

HRIGRU008267	WHITE VIENNA	Kohl rabi	Kohl rabi	G11	**IS**	**cultivar**	**S**	**DEL**	**I**	**A**	**S**	**S**	**P**	**N**	**K**	**DEL**	**A**	**Y**	**E**	**F**	**4**

HRIGRU005443	CAVOLO FORTE	Purple kohl rabi	Kohl rabi	G12	**ITA**	**cultivar**	**S**	**DEL**	**I**	**A**	**S**	**S**	**P**	**N**	**K**	**DEL**	**A**	**C**	**E**	**F**	**6**

HRIGRU007543	CHINESE KALE	Chinese kale	Alboglabra	A02	**CHINA**	**landrace**	**T**		**V**	**V**	**S**	**P**	**K**	**D**	**A**		**A**	**C**	**Q**	**F**	**1**

Senna (GK95186)	Senna	Chinese white kale	Alboglabra	A05	**N/A**	**DH**	**S**	**DEL**	**I**	**A**	**S**	**S**	**P**	**N**	**K**	**DEL**	**A**	**Y**	**E**	**F**	**4**

HRIGRU009490	COUVE CORTE	Tronchuda cabbage	Tronchuda cabbage	H02	**PORT**	**landrace**	**S**	**DEL**	**I**	**A**	**S**	**S**	**P**	**N**	**K**	**DEL**	**A**	**Y**	**E**	**F**	**4**

HRIGRU009574	COUVE PENCA DE GONDOMAR	Tronchuda cabbage	Tronchuda cabbage	H05	**PORT**	**landrace**	**T**		**V**	**V**	**S**	**P**	**K**	**D**	**A**		**A**	**C**	**Q**	**F**	**1**

HRIGRU007796		Wild cabbage	Wild cabbage	H08	**UK**	**N/A**	**S**	**DEL**	**I**	**A**	**S**	**S**	**P**	**N**	**K**	**DEL**	**A**	**Y**	**E**	**F**	**4**

Details of amino acid polymorphisms in the 55 *B. oleracea *genotypes from the BolDFS genotypes [60] for the first 650 bp of *BoFRIa*. The number of genotypes in a *BoFRIa *allele type within each vegetable type is also listed

### Functional analysis of *BoFRIa *alleles in *A. thaliana*

**Figure 5:**
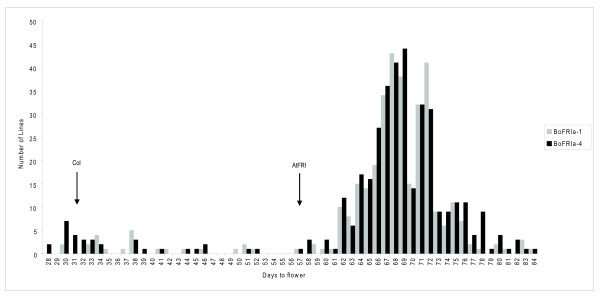
**Distribution of flowering times in T1 transformants carrying *BoFRIa-1 *and *BoFRIa-4 *alleles**. Histogram of the flowering time of T1 lines transformed with *BoFRIa-1 *and *BoFRIa-4 *measured as days to flower. The flowering time of wild type Col-0 plants and Col-0 transformed with *AtFRI *are indicated.

To ascertain if the two most common *BoFRIa *alleles conferred any functional differences we undertook transformation experiments. The coding and 3'UTR sequences from the *BoFRIa-1 *and *BoFRIa-4 *alleles were used to replace the *AtFRI *coding and 3' UTR sequences in an *A. thaliana *genomic clone. By retaining common regulatory sequences in the 5' region from the *AtFRI *gene we hoped to normalise expression and thus focus on the structural differences between the two *Brassica *proteins. These constructs were transformed into the rapid-cycling *A. thaliana *accession Columbia (Col-0). Col-0 carries a loss-of-function mutation within *AtFRI*, but has a functional *FLC *so these experiments would determine if *BoFRIa *could complement the *fri *mutation in Col-0, and induce late flowering. Both *BoFRIa *alleles complemented the loss-of-function mutation with > 100 primary (T1) transformed plants containing each of the *BoFRIa *alleles flowering very late compared to Col-0 plants and surprisingly also later than Col-0 transformed with a functional *AtFRI *(Figure 5).

**Figure 6:**
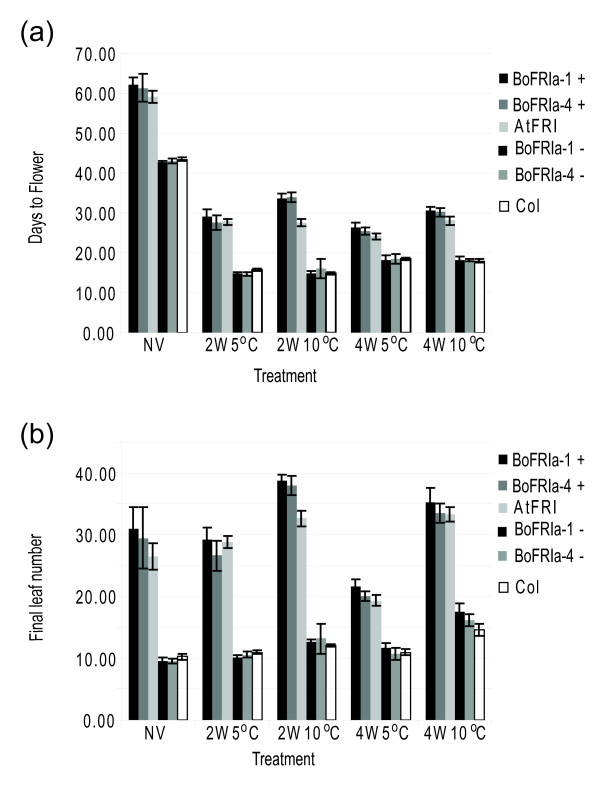
**Functional analysis of the two most common *BoFRIa *alleles**. Average flowering time of T2 families transformed with the two major *BoFRIa *alleles *BoFRIa-1 *and *BoFRIa-4 *and compared to Col-0 transformed with the *AtFRI *allele and Col-0 wild type. (a) Flowering time measured as days-to-flower. The error bars show 95% confidence intervals. (b) Flowering time measured as final leaf number. The error bars show 95% confidence intervals. Segregating progeny with and without the transgene are indicated by + and - respectively.

To investigate the functionality of *BoFRIa-1 *and *BoFRIa-4 *alleles under different environmental conditions five transformants carrying each allele were analysed in the next (T2) generation. Flowering time was analysed as days-to-flower and total leaf number in plants that had no vernalization, or had experienced two or four weeks vernalization, at either 5°C or 10°C (Figure 6). In all treatments, except two weeks at 10°C (2W10°C), plants with either *BoFRIa *allele flowered as late as those carrying *AtFRI*. At 2W10°C plants carrying either of the *BoFRIa *alleles flowered later than *AtFRI*. Figure 6 also shows that plants undergoing a vernalization treatment at 10°C compared to 5°C continue to grow and initiate leaves at a faster rate. Thus, when considering total leaf number as a measure of flowering time it appears that only 4W5°C was an effective vernalization treatment (Figure 6).

Expression of the coding sequences of the two *BoFRIa *alleles under the *AtFRI *5' regulatory sequences showed that both alleles can produce functionally equivalent proteins that may, under some environmental conditions, be even stronger with respect to flowering time effects than the endogenous *A. thaliana *protein (Figure 6). In contrast, two *A. lyrata FRI *alleles conferred a quantitative difference in flowering time by both association and transgenic studies [16]. The maintenance of both *A. lyrata *alleles at intermediate frequencies in natural populations suggests they are differentially selected in different environments. If the *BoFRIa *alleles do underlie flowering time QTL then there must be expression differences between the two genes to account for the difference in flowering time. Both these genes could be expressed in a very different pattern to *AtFRI *as the rearrangement that moved it to chromosome 4 resulted in completely different 5' sequences less than 1 kb upstream of the transcription start site and places it in a very different chromatin context since it is now 200-300 kb downstream of the heterochromatic nucleolar organizer region NOR4 [61].

## Conclusions

Knowledge of *B. oleracea FRI *gene number, functionality and map position now puts us in a strong position to undertake an extensive investigation into the contribution of allelic variation at *FRI *to flowering, vernalization and life history behaviours. Differences in life history between *A. lyrata *and *A. thaliana *such as outcrossing versus selfing and a perennial compared to annual habit may result in a requirement for some level of *FRI *functionality in *A. lyrata *that is optional in *A. thaliana *[16]. *B. oleracea*, like *A. lyrata*, is a largely outcrossing species and some wild *B. oleracea*, thought to be the progenitor of the modern crop plants, have been reported to keep flowering for up to 20 years [62]. Our analysis of *BoFRIa *suggests that only a small number of functional *BoFRIa *alleles are captured within the cultivated *B. oleracea *germplasm. To date we have found no evidence for loss-of-function mutations that are frequent in *AtFRI*. Further analysis of the 5' and 3' regulatory regions of *BoFRI *is now underway. The proximity of *BoFRI *to *BoFLC, BoFY *and *BoCO *opens up new questions of how this may influence flowering behaviour. It will be particularly important to be in a position to select specific alleles in breeding programmes to allow us to enhance robustness against increasing climate variability.

## Methods

### Cloning *BoFRI *genes

The JBo BAC library was hybridised with the *AtFRI *genomic clone, originally from the accession Stockholm [8] and seven BACs identified, six having identical restriction patterns and one different. Purified genomic DNA was prepared (Qiagen Maxi Prep Kit) from two of these BACs (72I23 and 88G16) and used to generate shotgun libraries (TOPOr Shotgun Kit) of 1-2 kb fragments, in the pCR^R^4Blunt-TOPO^R ^vector, giving 6-fold coverage. Colonies from these libraries were gridded onto nylon membrane (HyBond-N+) and hybridised to three probes generated from *AtFRI *(the 5' region, exon 1, and the 3' end of exon 3 and 3' UTR). BAC sub-clones were identified with each of these probes. Sequence analysis confirmed that the two BACs carried different *Brassica *paralogues.

### Mapping *BoFRI loci *in *B. oleracea *mapping populations

#### Genetic mapping

Mapping filters of the A12DHdxGDDH33 mapping population were produced and hybridized with a conserved *BoFRI *probe as described in [36,63]. A 900 bp conserved region from exons 2 and 3 from *BoFRIb *was amplified from A12DHd genomic DNA with primers J2NG_F1 (5' AAGTATCAAGCGTGGAAAGCA 3') and J2NG_R1 (5' GTTACGAGGAGACCTGTGATT 3') and used to probe both the A12DHdxGDDH33 and NxG mapping filters (supplied by Graham Teakle, WHRI). Linkage analysis to map the *BoFRIa *locus was performed using Joinmap 3.0 [64] with the mapping data provided at BrassicaDB http://brassica.bbsrc.ac.uk/BrassicaDB/.

#### Fluorescence in situ hybridisation (FISH)

FISH was performed on chromosome spreads from the A12DHd genotype of *B. oleracea *using methods described in [65]. The chromosomes are now named according to their corresponding linkage group. JBo72I23 was applied to meiotic pachytene spreads together with JBo62M08, a BAC which is associated with the RFLP marker pN22 on C3 at 42 cM and previously assigned to chromosome C3 by FISH. JBo88G16 (*BoFRIb*) was applied to mitotic metaphase spreads together with BAC BoB061G14, which hybridizes to pericentromeric heterochromatin of six pairs of chromosomes, and a 45S rDNA probe from clone pTa71 [66], EMBLX07841. The chromosome pair to which JBo88G16 hybridized lacked signals from the other probes and had morphology suggestive of C9. Therefore, slides were reprobed with JBo32J18, a BAC associated with *BoFLC1 *which has been mapped to a region between pN47E4NM (87 cM) and pN3E1 (103 cM) on C9 [31,67] and confirmed to be on C9 by FISH (unpublished). Two further BACs showing the same restriction pattern as JBo88G16 were applied separately with JBo88G16 to pachytene spreads.

### Sequencing *BoFRI*a in BolDFS

The *B. oleracea *diversity foundation set (BolDFS) is a core collection of lines that represent the genetic variation across the morphologically diverse crops of this species http://www.brassica.info/resource/plants/diversity_sets.php. DNA was isolated using the DNeasy 96 Plant Kit (Qiagen) and amplified using the GenomiPhi whole genome amplification kit (GE healthcare). A 650 bp fragment of *BoFRIa *was amplified from genomiphied DNA of 55 genotypes of the BolDFS by PCR with primers YWFRI_F (5'CGCACATCGTCCATCAACAAG 3') and FRIJ1_R2 (5'ATCCTTCACCCACCAGCCT 3') using AMPLITAQ GOLD TAQ DNA Polymerase (Life Technologies Ltd (Invitrogen Division)). Sequence analysis was conducted using AlignX in Vector NTI (Invitrogen).

### Functional analysis of *BoFRIa *alleles

Plasmid pFRIg (in pBluescript-KS+, Stratagene) was mutagenised to introduce a *Bam*HI site immediately 5' of the ATG (plasmid pFRIg-B). Digestion of pFRIg-B with *Bam*HI plus *Cla*I allowed removal of the *AtFRI *coding sequences, leaving the 5' region of *AtFRI*. A 4.3 kb fragment containing *BoFRIa *was isolated from genomic DNA of lines E1 and E8 by PCR with primers BoFRI1_Bam_ATG (5'CTTCCGCGGATCCCATGGCCGTCCGTAAC3') and BoFRI1_R2_ClaI (5'CAGAGATCGATCTCGAGAAAGGTAGCTGTTT 3'), using *PfuUltra *II Fusion HS DNA Polymerase (Agilent Technologies) and sequenced. PCR products were digested with *Bam*HI plus *Cla*I and the purified fragments ligated into *Bam*HI plus *Cla*I-digested pFRIg-B to give final constructs containing the 5'UTR of *AtFRI *with the coding and 3' UTR sequences of *BoFRIa*-1 (in BoFRIa-1) or *BoFRIa-4 *(in BoFRIa-4). pFRIg-B was used as the *A. thaliana FRI *control. The final constructs were ligated into binary vector pSLJ755I6 (a gift from Prof. Jonathan Jones, http://www.tsl.ac.uk/research/jonathan-jones/plasmids.htm), on an *Eco*RI plus *XhoI *fragment (pFRIg-B) or *Eco*RI plus *Cla*I fragments (from BoFRIa-1 and BoFRIa-4). The constructs were transferred into *Agrobacterium *by triparental mating [68] and transformed into *A. thaliana *accession Col-0 by a floral dipping method (modified from [69]). T1 transformants were isolated by selection for Basta^™ ^resistance. T2 seed were collected and flowering time determined by days-to-flower excluding the period of vernalization treatment and final leaf number at flowering.

### Plant growth

T2 *A. thaliana *seeds were sown on 'Arabidopsis mix' (Scotts^® ^Levington F2 8.75 l bags,100 l of grit, 200 g of Imidasect^® ^5 gr.) in plastic pots (7 cm × 7 cm) and stratified in a vernalization chamber at 5°C with an 8 h photoperiod and constant humidity for 3 days. Pots were moved to a naturally lit long day glasshouse for 7 days in May 2010 to allow germination and pre-growth. Seedlings not receiving a vernalization treatment remained in the glasshouse; seedlings to be vernalization treated were transferred back to a vernalization room or controlled environment cabinet (Snijder Economic Deluxe) for a treatment of either two or four weeks at 5°C or 10°C. After vernalization, 20 plants per line were transplanted into trays with 40 cells of 2 cm × 2 cm and returned to the glasshouse. Trays were moved regularly to random positions to prevent any positional effects on plant growth. Flowering time was recorded as either total leaf number (rosette leaves plus cauline leaves at flowering) or bolting time; bolting time was scored as the number of days-to-flowering determined when the inflorescence stem was 3 cm tall.

## Abbreviations

QTL: Quantitative trait loci; BAC: Bacterial artificial chromosome; ORF: open reading frame; FISH: fluorescence in situ hybridization.

## Authors' contributions

JI and CD conceived and designed the experiments, supervised the work and wrote the paper. CL, YZ and JI analyzed the BoFRIa alleles. ES and JI analyzed the BolDFS and GT contributed DNA from the BolDFS. EH performed the FISH experiments. All authors approved and read the final manuscript.
